# The effect of mobile phone task and age on gait: A systematic review and meta-analysis

**DOI:** 10.3389/fphys.2023.1163655

**Published:** 2023-04-04

**Authors:** Xinxin Zhang, Qiang Li, Pincao Gao, Jialin Zhu, Haowen Tuo, Qihan Lin, Feiyue Jing, Weiguo Liu

**Affiliations:** ^1^ College of Physical Education and Health, Guangxi Normal University, Guilin, China; ^2^ Department of Physical Education, Ocean University of China, Qingdao, China; ^3^ College of Rehabilitation and Health, Hunan University of Medicine, Huaihua, China

**Keywords:** mobile phone use, age, gait, walking safety, meta-analysis

## Abstract

**Objective:** Mobile phone usage while performing postural-locomotor tasks is everyday activity across persons of all ages in various environmental contexts and health conditions. However, it is also an important factor contributing to accidents. To lower the risk of pedestrian accidents, this meta-analysis aimed to examine how mobile phones affect pedestrian gait and identify how mobile phone tasks and participant age affect gait differently.

**Methods:** Electronic database searches were performed in The Cochrane Library, PubMed, and Medline. Two examiners evaluated the eligibility and quality of included studies using the Downs and Black checklist. The mean differences (MD) or standardized mean differences (SMD) were calculated for each outcome. Subgroup analyses were used to compare the differential effects of mobile phone task and participant age on gait.

**Results:** Among 22 eligible studies, 592 participants in 10 countries were analyzed in this meta-analysis. The overall meta-analysis showed that using a mobile phone significantly decreased pedestrian gait velocity (SMD = −1.45; 95% CI: −1.66 to −1.24; *p* < 0.00001; I^2^ = 66%), step length (SMD = −1.01; 95% CI: −1.43 to −0.59; *p* < 0.00001; I^2^ = 82%), and stride length (SMD = −0.9; 95% CI: −1.19 to −0.60; *p* < 0.00001; I^2^ = 79%), significantly increased pedestrian step time (SMD = 0.77; 95% CI: 0.45 to 1.08; *p* < 0.00001; I^2^ = 78%), stride time (SMD = 0.87; 95% CI: 0.69 to 1.06; *p* < 0.00001; I^2^ = 24%), step width (SMD = 0.79; 95% CI: 0.34 to 1.24; *p* = 0.0006. I^2^ = 75%), double support time (SMD = 1.09; 95% CI: 0.86 to 1.31; *p* < 0.00001; I^2^ = 42%), and double support (%gait cycle, %GC) (MD = 2.32; 95% CI: 1.75 to 2.88; *p* < 0.00001; I^2^ = 26%).

**Conclusion:** In summary, the effects of mobile phone tasks and participant age on gait were inconsistent. Our study found that resource-intensive tasks (texting and reading) significantly reduced gait velocity, and step time; however, small resource-intensive tasks (calling, talking, and dialing) did not affect these outcomes. In contrast to young adults, step length and step time were not affected by mobile phone use in older adults. Tips: Pedestrians should consider using a mobile phone in their daily lives according to the application scenarios (walking environment, the complexity of mobile phone tasks, pedestrians’ task processing abilities, etc.) as appropriate to avoid dangerous accidents.

**Systematic Review Registration:** identifier CRD42022358963.

## Introduction

As communication technology progresses, smartphones have become essential for our social life, entertainment, and education (Galván et al., 2013) and have become an indispensable component of people’s life (Krasovsky et al., 2017). People used mobile phones for around 2.3 trillion minutes in 2013, with most of the usage scenarios taking place in public places (Galván et al., 2013). In 2018, nine countries worldwide had a mobile phone usage coverage of more than 70% (Kim et al., 2020), with more than 25 million Americans using smartphones (Kim et al., 2020). Both the number of smartphone users and their usage rates are increasing. While mobile phone usage has brought significant convenience to our lives, the number of accidents caused by inappropriate mobile phone use is rising (Demura and Uchiyama, 2009). According to a study (Nasar and Troyer, 2013) conducted by the National Electronic Equipment Injury Surveillance System (NEISS), the proportion of pedestrian accidents due to mobile phone usage grew from 0.58 percent in 2004 to 3.67 percent in 2010, an almost tenfold rise.

Walking is a highly automated task that effectively integrates cognitive, proprioceptive, and feedback systems (Adolph and Robinson, 2013), and each stage of its execution requires the involvement of cognitive resources and is challenged by postural control (Fahyan, 2010). Using a mobile phone while walking is a typical dual-task paradigm (Plummer et al., 2013), meaning that participants must perform mobile phone operations and walking tasks simultaneously. Based on the theory of finite capacity scheduling, the state of the various tasks outcomes how much cognitive or motor performance is impacted (Coker, 2017; Wickens and Damos, 2020).

Compared to performing a single task such as arithmetic, language, memory, and motor while walking, the mobile phone task is more like integrating multiple single tasks. Its demand for cognitive resources is significantly higher than other simple tasks (Tan et al., 2022). Previous studies have shown that the dual-task paradigm of using a mobile phone while walking is more likely to cause a reduced perception of the surroundings, reduced motor function of the lower limbs, and distraction than other simple tasks while walking in pedestrians (Kim et al., 2020; Tan et al., 2022). Using mobile phone have adverse effects on pedestrian visual information, motor control, and motor responses, resulting in altered gait parameters (Strayer et al., 2003; Lee and Strayer, 2004; McPhee et al., 2004; Kim et al., 2017; Ortiz et al., 2018; Tan et al., 2022).

As traffic conditions become more complex, exploring the impact of mobile phone use on pedestrians will be necessary for pedestrian walking safety. However, the effects of mobile phones on pedestrians are still unclear. There are some differences in the results of previous studies (Kao et al., 2015; Lim et al., 2015; Magnani et al., 2017), particularly the influence of different mobile phone operating tasks and the age of the participant population on the outcomes of the studies (Jeon et al., 2015; Kao et al., 2015; Strubhar et al., 2015; Crowley et al., 2019; Crowley et al., 2021). In addition, although previous meta-analyses were conducted, they were only explored for different ages and did not examine different mobile phone operation tasks (Bruyneel et al., 2023). Therefore, to lower the risk of pedestrian accidents and to inform future studies. This meta-analysis aimed to review the available literature, analyze the effects of mobile phone usage on gait comprehensively, and perform subgroup analyses to identify how mobile phone tasks and participant age affect gait differently.

## Methods

This review followed with the Preferred Reporting Items for Systematic Reviews and Meta-Analyses (PRISMA) statement (Moher et al., 2009), and was registered in the International Prospective Register of Systematic Reviews (PROSPERO) with the identifier: CRD42022358963. Since this study was a systematic review, human ethics committee approval was unnecessary.

### Study search and selection

The Cochrane library, PubMed, and Medline databases were searched for references up to August 2022 using English as the only acceptable language. Search terms included: (a) Mobile Phone or Cell Phone or Smartphone or Phone or Moblie phone use and (b) Gait or Walking or Postural balance (Related to step width while walking). The inclusion criteria of this meta-analysis included: (a) The intervention was to use the phone while walking. Regarding mobile phone characteristics, no distinction was made between the operating system (e.g., Apple, Android), phone type (e.g., brands, versions), (b) reporting quantitative data related to gait, outcomes mainly including Gait velocity, Stride length, Stride time, Step length, Step time, Step width, Double support (%gait cycle, % GC), Double support time, and Cadence, etc., (c) The studies were required to provide a comparison, with the walking task in single-task and mobile phone task conditions being similar, and (d) only peer-reviewed, original, and cross-sectional observational studies were included. Studies were excluded if they lacked a control group or if outcome data were insufficiently supplied. Also omitted were review articles, editorials, and conferences.

EndNote X9 was used to eliminate duplicates from the search, and then two reviewers (XZ and PG) independently assessed the titles and abstracts of articles to establish their appropriateness for inclusion. Studies that did not meet the inclusion criteria were not considered further. Those that could not be eliminated were retrieved, and the full text was reviewed by two persons (XZ and PG). When data confirmation or further information was requested, the authors were contacted *via* email. Disagreements or misunderstandings were resolved through a conversation with a third reviewer (WL).

### Data extraction and quality assessment

The included studies collected the following information: first author, country, year of publication, characteristics of the participants, and specific details of experimental design, such as mobile phone intervention tasks, experimental environment, and outcome measurements. On the other hand, since both variables, cadence and stride/step time, illustrate the same temporal aspect of gait, the only difference is that one is the inverse of the other. Therefore, the cadence outcome was transformed into stride or step time based on the raw results (strides/min or steps/min) during the outcome data extraction.

Due to the observational cross-sectional design of the included studies, many usual tools for evaluating the quality of randomized controlled trials were unsuitable. Therefore, the Downs and Black checklist was used to assess the quality of included studies (Downs and Black, 1998). This instrument is appropriate for all quantitative study designs (Bruyneel et al., 2023). The quality assessment had five subscales: reporting (items 1–8), external validity (items 9–11), internal validity (items 12–15), and power (item 16). “Yes” received a score of 1 for all items, while “no” and “unable to decide” received scores of 0. A total score (/16 points) was calculated for each study. The disagreements between the two evaluators (XZ and PG) were addressed through discussion with a third reviewer (WL).

### Data synthesis and statistical analysis

This meta-analysis was performed using RevMan 5.2 and Stata 14.0 software. Because the outcome parameters of all studies were continuous variables, we used mean difference (MD) or standardized mean difference (SMD) as effect sizes and calculated 95% confidence intervals (CI) for the combined results. The SMD was used when the same outcome indicator was measured in different units (Bruyneel et al., 2023). The MD and SMD absolute values of 0.2 represent a small effect, 0.5 a moderate effect, and 0.8 a large effect (Cohen, 1988). Study heterogeneity was examined using the Chi^2^ test and the I^2^ statistic. If the heterogeneity test did not demonstrate statistical significance (I^2^ <50%; *p* > 0.05), the fixed-effects model was applied. Apart from that, a random-effects model was applied (Higgins et al., 2003). Subgroup and sensitivity analyses were conducted to explore the sources of heterogeneity for outcome indicators with heterogeneity. Publication bias was evaluated using an Egger asymmetry test, and the effect of publication bias on the results was evaluated using the trim and fill method. Statistically significant differences were set at α = 0.05.

## Results

### Search results

**FIGURE 1:**
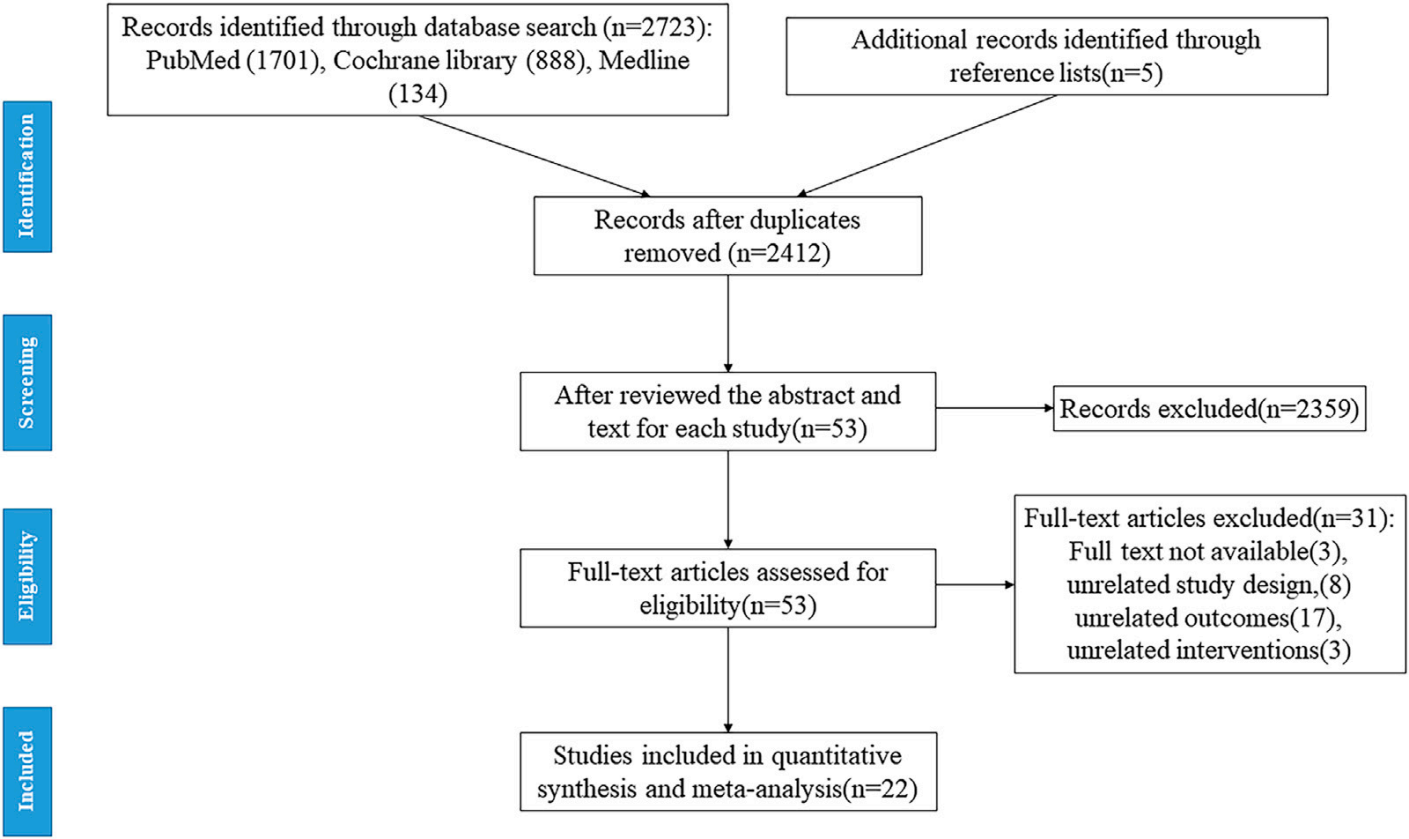
The flow diagram of the selection process.

A total of 2,732 records were collected, and 2,415 records were included in the preliminary screening after EndNote was used to eliminate duplicates. Based on our inclusion and exclusion criteria, three reviewers looked at the abstract and title of each study. They excluded 2,359 studies because of unrelated research topics, study design, outcome parameters, and review articles. Fifty-three records entered full-text screening. Finally, 22 studies (Demura and Uchiyama, 2009; Schabrun et al., 2014; Agostini et al., 2015; Jeon et al., 2015; Kao et al., 2015; Licence et al., 2015; Lim et al., 2015; Plummer et al., 2015; Strubhar et al., 2015; Seymour et al., 2016; Magnani et al., 2017; Krasovsky et al., 2018; Lee and Lee, 2018; Pau et al., 2018; Sirhan et al., 2018; Crowley et al., 2019; Feld and Plummer, 2019; Prupetkaew et al., 2019; Kim et al., 2020; Crowley et al., 2021; Krasovsky et al., 2021; Tandon et al., 2021) met the inclusion criteria. The flowchart is shown in Figure 1.

## Included studies’ characteristics

The 22 studies included in the Meta-analysis were all observational cross-sectional designs. They included a total of 592 participants from 10 countries, including seven studies from the USA (Kao et al., 2015; Licence et al., 2015; Lim et al., 2015; Plummer et al., 2015; Strubhar et al., 2015; Seymour et al., 2016; Feld and Plummer, 2019), three from Israel (Krasovsky et al., 2018; Sirhan et al., 2018; Krasovsky et al., 2021), and Korea (Jeon et al., 2015; Lee and Lee, 2018; Kim et al., 2020), two from Italy (Agostini et al., 2015; Pau et al., 2018), and Denmark (Crowley et al., 2019; Crowley et al., 2021), and one from Japan (Demura and Uchiyama, 2009), Australia (Schabrun et al., 2014), Brazil (Magnani et al., 2017), Thailand (Prupetkaew et al., 2019), and UK (Tandon et al., 2021). Of the included studies, four studies (Kao et al., 2015; Seymour et al., 2016; Krasovsky et al., 2018; Prupetkaew et al., 2019) tested mobile phone interventions for older people, two studies (Krasovsky et al., 2018; Prupetkaew et al., 2019) performed comparative analyses for different experimental settings, and nine studies (Kao et al., 2015; Licence et al., 2015; Plummer et al., 2015; Strubhar et al., 2015; Seymour et al., 2016; Krasovsky et al., 2018; Pau et al., 2018; Feld and Plummer, 2019; Krasovsky et al., 2021) did not report on participant height or weight. For testing cell phone tasks, 19 studies used the texting tasks as an intervention (Demura and Uchiyama, 2009; Schabrun et al., 2014; Agostini et al., 2015; Licence et al., 2015; Lim et al., 2015; Plummer et al., 2015; Strubhar et al., 2015; Magnani et al., 2017; Krasovsky et al., 2018; Lee and Lee, 2018; Pau et al., 2018; Sirhan et al., 2018; Crowley et al., 2019; Feld and Plummer, 2019; Prupetkaew et al., 2019; Kim et al., 2020; Crowley et al., 2021; Krasovsky et al., 2021; Tandon et al., 2021), reading (Schabrun et al., 2014; Krasovsky et al., 2021), dialing (Kao et al., 2015; Seymour et al., 2016), calling (Magnani et al., 2017; Crowley et al., 2021), web browsing (Jeon et al., 2015) and music listening tasks (Magnani et al., 2017) were also explored as interventions, respectively. On the other hand, in terms of outcome indicators, most studies (Demura and Uchiyama, 2009; Schabrun et al., 2014; Agostini et al., 2015; Jeon et al., 2015; Licence et al., 2015; Plummer et al., 2015; Strubhar et al., 2015; Krasovsky et al., 2018; Pau et al., 2018; Sirhan et al., 2018; Crowley et al., 2019; Feld and Plummer, 2019; Prupetkaew et al., 2019; Kim et al., 2020; Crowley et al., 2021; Krasovsky et al., 2021) (72.73%) explored the gait velocity indicator. Few studies (18.18%) performed step time (Kao et al., 2015; Sirhan et al., 2018; Prupetkaew et al., 2019; Tandon et al., 2021) and double support time (Jeon et al., 2015; Licence et al., 2015; Lim et al., 2015; Kim et al., 2020) indicator analyses. Specific details regarding included studies’ characteristics are shown in Table 1.

**TABLE 1:** The detailed characteristics of each included study.

First author	Years	Country	Sample	Sex (male/women)	Age (male/women)	Height (m)	Mass (kg)	Environment	Intervention	Control	Outcomes
Demura, Shinichi	2009	Japan	30	15/15	20.3 ± 0.98/19.4 ± 0.83	1.66 ± 0.08	62.95 ± 7.98	L	texting	Only walking	①, ②, ⑥
Schabrun, S. M	2014	Australia	26	7/19	29 ± 11	1.7 ± 0.1	71 ± 13	L	reading; texting	Only walking	①, ②, ③
Agostini, V	2015	Italy	18	8/10	20–30	1.69 ± 0.08	63.3 ± 10	L	texting	Only walking	①, ②, ③
											⑦
Jeon, S	2015	Korea	26	16/10	21.73	1.70	62.12	L	browsing, browsing + calling	Only walking	①, ②, ④
											⑤, ⑧
Kao, P. C	2015	USA	Y: 7; O: 9	Y: 2/5; O: 2/7	Y: 20.4 ± 2.2; O: 61.1 ± 10.0	NR	NR	L	dialing	Only walking	④, ⑤, ⑥
Licence, S	2015	USA	30	12/18	18–50	NR	NR	L	texting	Only walking	①, ④, ⑤, ⑧
Lim, J	2015	USA	20	10/10	20.3 ± 1.2	1.7 ± 0.1	69.8 ± 13.7	L	texting	Only walking	②, ③, ⑥, ⑧
Plummer, P	2015	USA	32	12/20	22.5 ± 2.1	NR	NR	L	texting	Only walking	①
Strubhar, A. J	2015	USA	32	6/26	18–40	NR	NR	L	texting	Only walking	①, ④, ⑤, ⑦
Seymour, K. M. (Seymour et al., 2016)	2016	USA	Y: 11; O: 11	Y: 4/7; O: 3/8	Y: 20.1 ± 1.9; O: 60.5 ± 9.4	NR	NR	L	dialing	Only walking	②, ⑥
Magnani, R. M	2017	Brazil	20	10/10	24.5 ± 3.3	1.62 ± 36.7	69.0 ± 13.7	L	talking, texting, listening	Only walking	④, ⑤, ⑥
Krasovsky, T	2018	Israel	Y: 30; O: 20	Y: 15/15; O: 13/7	Y: 27.8 ± 4.4; O: 68.9 ± 3.9	Y: 1.71 ± 0.1; O: 1.65 ± 0.08	NR	L, R	texting	Only walking	①, ②, ③
Lee, J. H	2018	Korea	39	19/20	22.26 ± 0.27	1.69 ± 0.01	63.31 ± 1.81	L	texting	Only walking	⑥
Pau, M	2018	Italy	40	11/29	41.3 ± 10.4	1.65 ± 0.08	NR	L	texting	Only walking	①, ②, ⑤, ⑦
Sirhan, B. (Sirhan et al., 2018)	2018	Israel	15	7/8	37.4 ± 6.3	1.71 ± 0.08	73.4 ± 13.5	L	texting	Only walking	①, ②, ⑤
Crowley, P. (Crowley et al., 2019)	2019	Denmark	10	7/3	24.7 ± 4.4	1.76 ± 0.05	71.9 ± 12.2	L	texting	Only walking	①, ②, ⑤, ⑦
Feld, J. A	2019	USA	20	9/11	25.0 ± 2.8	NR	NR	L	texting	Only walking	①
Prupetkaew, P	2019	Thailand	Y: 12; O: 12	Y: 4/8; O: 2/10	Y: 22.7 ± 1.8; O: 73.5 ± 5.6	Y: 1.61 ± 0.07; O: 1.58 ± 0.06	Y: 56.7 ± 9.9; O: 59.3 ± 7.2	L, R	texting	Only walking	①, ④, ⑤
Kim, S. H	2020	Korea	36	26/10	24.69 ± 1.94	1.72 ± 0.08	65.66 ± 12.56	L	texting	Only walking	①, ②, ④,⑤
											⑦, ⑧
Crowley, P	2021	Denmark	20	11/9	27 ± 5.5	1.74 ± 0.07	71 ± 10.6	L	texting, talking	Only walking	①
Krasovsky, T	2021	Israel	29	14/15	26 ± 4.18	1.69 ± 0.1	NR	L	texting, reading	Only walking	①, ②, ③
Tandon, R. (Tandon et al., 2021)	2021	UK	37	13/24	22.53 ± 5.83	1.71 ± 0.1	67.78 ± 15.95	L	texting	Only walking	⑤

Notes: Y, younger; O, older; NR, not reported; L, performing experimental tests in the laboratory; R, performing experimental tests in the street; ①, Gait velocity; ②, Stride length; ③, Stride time; ④, Step length; ⑤, Step time; ⑥, Step width; ⑦, Double support (% GC); ⑧, Double support time.

### Methodological quality assessment

The quality scores were between 8 and 10 points/16 for twelve studies (Demura and Uchiyama, 2009; Schabrun et al., 2014; Kao et al., 2015; Licence et al., 2015; Lim et al., 2015; Strubhar et al., 2015; Seymour et al., 2016; Magnani et al., 2017; Lee and Lee, 2018; Feld and Plummer, 2019; Crowley et al., 2021; Tandon et al., 2021), between 11 and 12/16 for eight studies (Agostini et al., 2015; Jeon et al., 2015; Krasovsky et al., 2018; Pau et al., 2018; Crowley et al., 2019; Prupetkaew et al., 2019; Kim et al., 2020; Krasovsky et al., 2021), and superior to 12/16 for two studies (Plummer et al., 2015; Sirhan et al., 2018). All included studies provided accurate main outcomes (valid and reliable). The most obvious problems involved external validity, selection bias, and power (e.g., determining if participants and places were representatives of the whole population, whether there was an adequate adjustment for confounding in the studies from which the primary findings were drawn, and whether the sample size was sufficient) (Table 2).

**TABLE 2:** Methodological quality assessment for inclusion in the study according to downs and black checklist.

First author and years	Demura, Shinichi 2009	Schabrun, S. M. 2014	Agostini, V. 2015	Jeon, S. 2015	Kao, P. C. 2015	Licence, S. 2015	Lim, J. 2015	Plummer, P. 2015	Strubhar, A. J. 2015	Seymour, K. M. 2016	Magnani, R. M. 2017	Krasovsky, T. 2017	Lee, J. H. 2018	Pau, M. 2018	Sirhan, B. 2018	Crowley, P. 2019	Feld, J. A. 2019	Prupetkaew, P. 2019	Kim, S. H. 2020	Crowley, P. 2021	Krasovsky, T. 2021	Tandon, R. 2021
Reporting																						
Q1	Y	Y	N	Y	Y	Y	Y	Y	Y	Y	Y	Y	Y	Y	Y	Y	N	Y	N	N	Y	N
Q2	Y	N	Y	N	Y	Y	Y	Y	Y	Y	Y	Y	Y	Y	Y	Y	Y	Y	Y	Y	N	Y
Q3	Y	Y	Y	Y	Y	N	Y	Y	Y	Y	Y	Y	Y	Y	Y	Y	N	Y	Y	Y	Y	N
Q4	Y	Y	Y	Y	Y	Y	Y	Y	Y	Y	Y	Y	Y	Y	Y	Y	Y	Y	Y	N	Y	Y
Q5	U	Y	Y	Y	N	N	Y	Y	Y	Y	Y	Y	N	Y	Y	Y	Y	Y	Y	Y	Y	Y
Q6	Y	Y	Y	Y	Y	Y	N	Y	Y	Y	Y	Y	Y	Y	Y	Y	Y	Y	Y	Y	Y	Y
Q7	U	Y	Y	Y	Y	Y	Y	Y	Y	Y	N	Y	Y	Y	Y	Y	Y	Y	Y	Y	Y	Y
Q8	Y	Y	Y	Y	Y	Y	N	Y	Y	N	Y	N	N	Y	Y	Y	N	Y	Y	N	Y	N
External validity																						
Q9	Y	Y	N	Y	U	Y	Y	N	Y	Y	Y	Y	Y	Y	Y	N	Y	Y	N	N	Y	Y
Q10	U	U	Y	Y	U	U	U	Y	N	N	U	U	U	Y	Y	N	Y	N	N	U	N	Y
Q11	N	N	N	Y	Y	N	N	Y	N	N	U	Y	N	N	U	N	N	Y	N	Y	N	N
Internal validity																						
Q12	Y	Y	Y	Y	Y	Y	Y	N	N	Y	Y	Y	Y	N	Y	Y	Y	Y	Y	Y	Y	Y
Q13	Y	Y	Y	Y	Y	Y	Y	Y	Y	Y	Y	Y	Y	Y	Y	Y	Y	Y	Y	Y	Y	Y
Internal validity (confounding) (selection bias)																						
Q14	N	U	Y	N	N	U	N	Y	U	N	U	U	N	Y	Y	U	U	U	Y	U	Y	U
Q15	N	N	Y	N	N	N	N	Y	N	N	N	N	N	N	N	Y	N	N	Y	N	N	N
Power																						
Q16	N	N	N	N	N	N	N	Y	N	N	N	N	N	N	N	N	N	N	N	N	Y	N
Total score																						
Score/16	9	10	12	12	10	9	9	14	10	10	10	11	9	12	13	11	9	12	12	8	12	9

Abbreviations: Y, yes; N, no; U, unable to decide.

### Meta-analysis results

**FIGURE 2:**
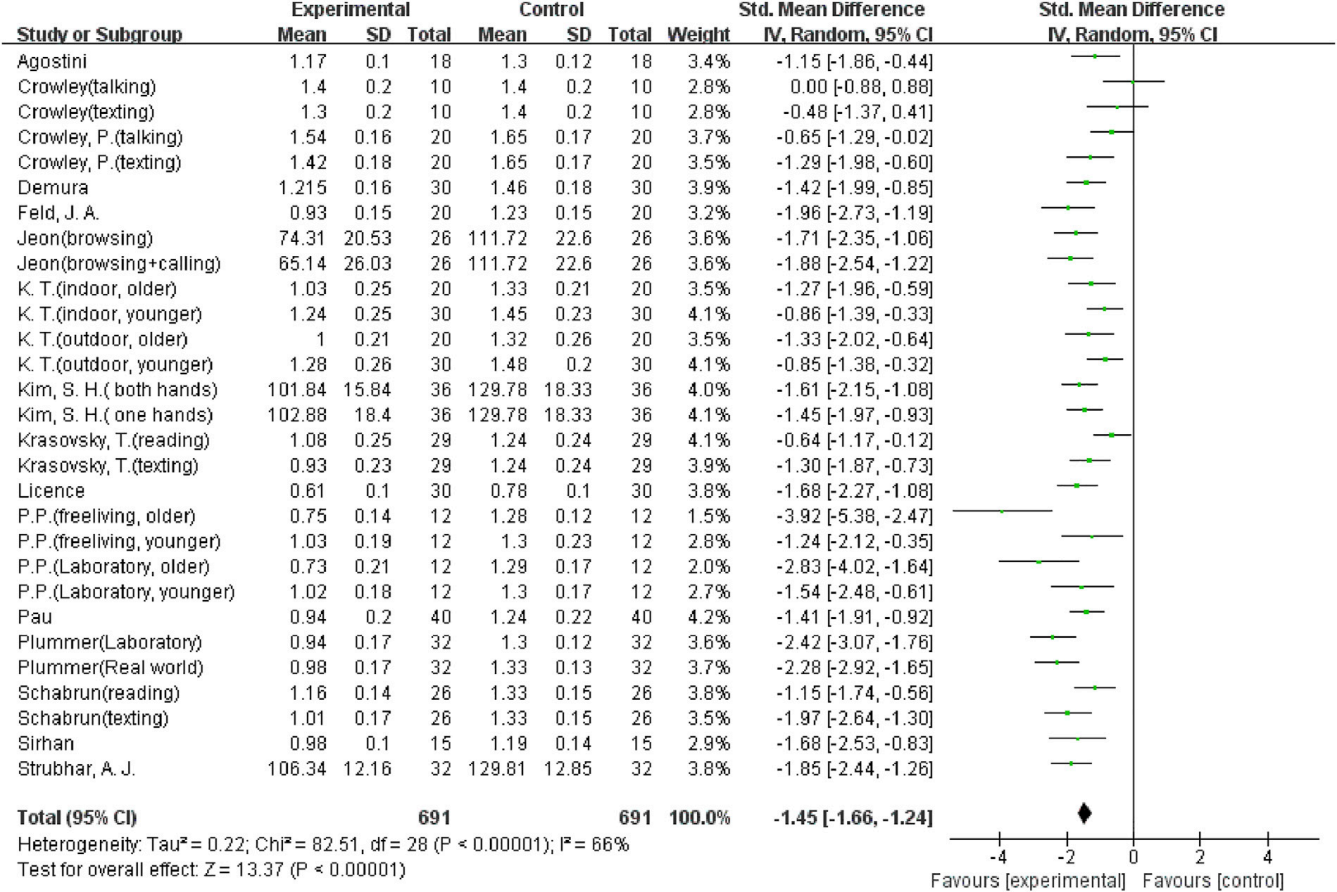
The effect of mobile phone use on gait velocity.

**FIGURE 3:**
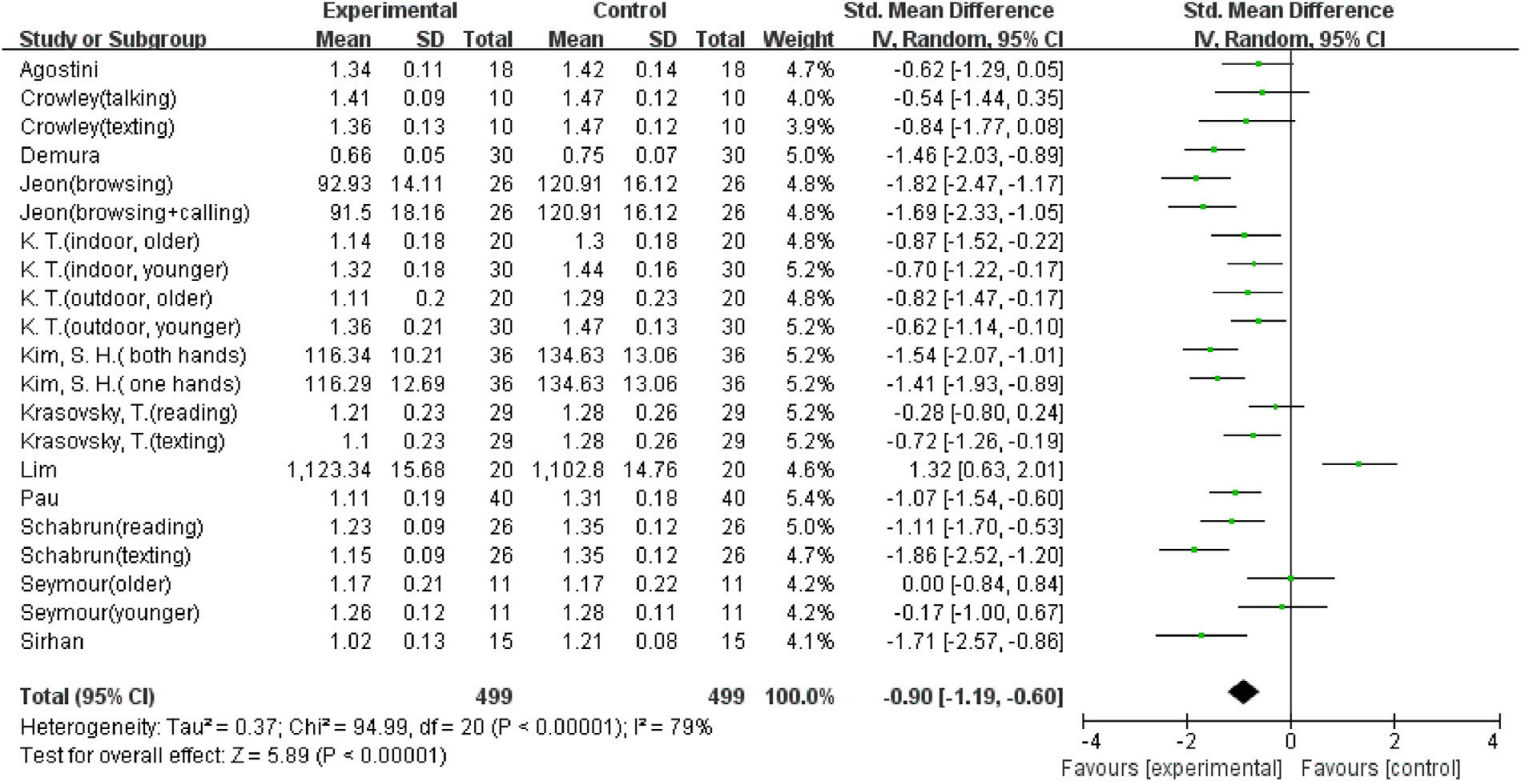
The effect of mobile phone use on stride length.

**FIGURE 4:**
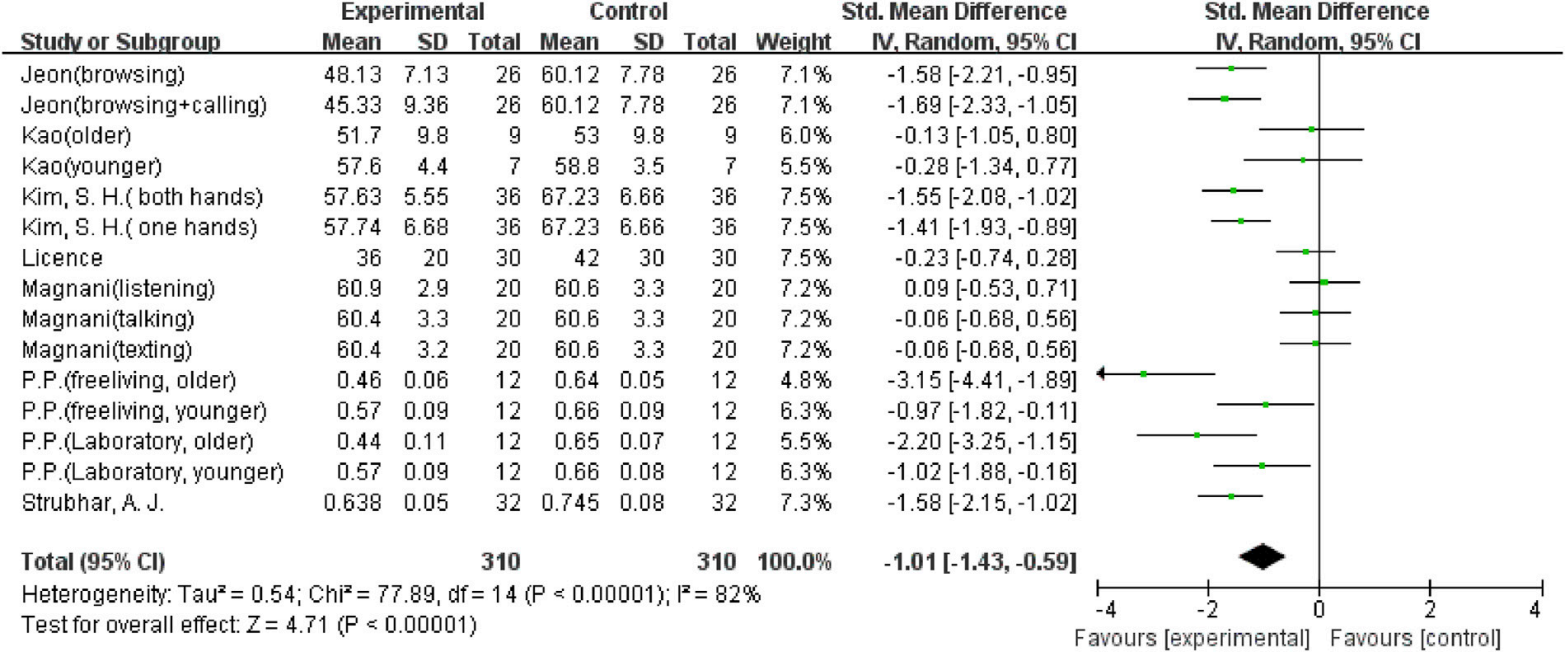
The effect of mobile phone use on step length.

**FIGURE 5:**
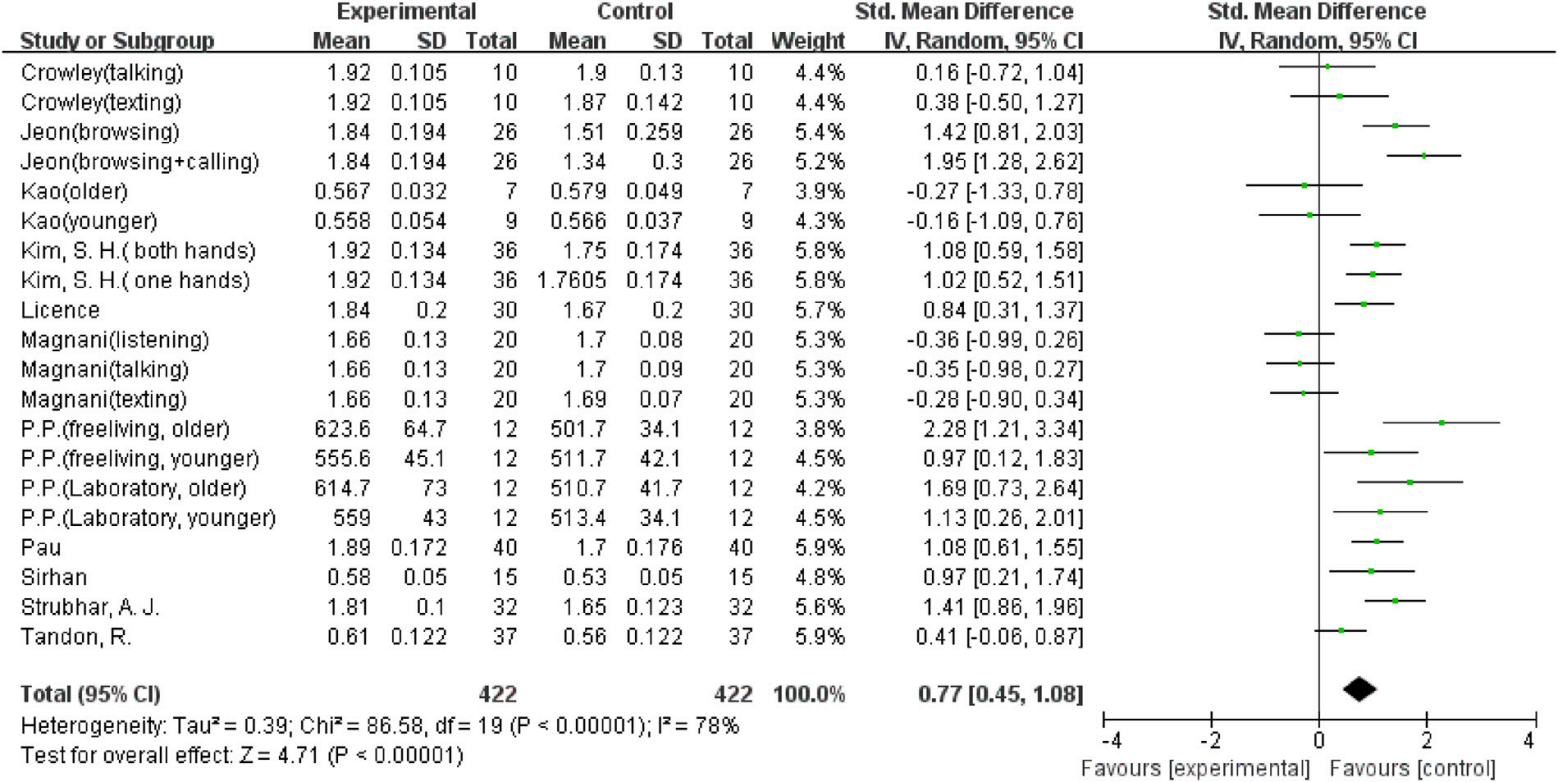
The effect of mobile phone use on step time.

**FIGURE 6:**
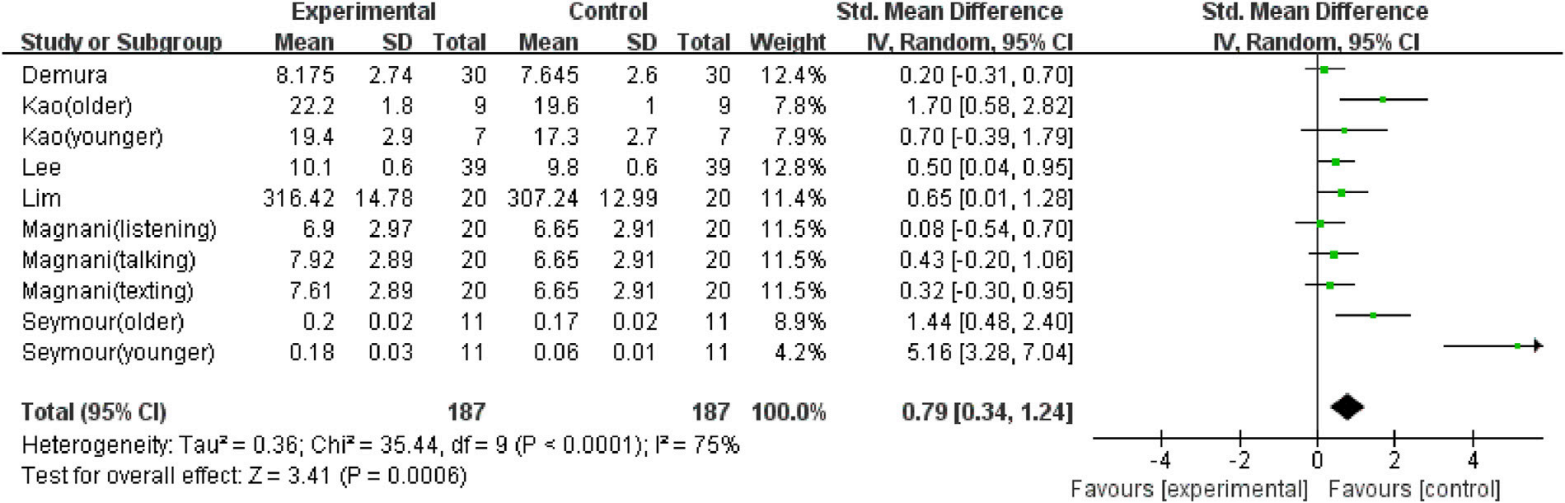
The effect of mobile phone use on step width.

The overall meta-analysis results showed considerable heterogeneity in the five outcomes (gait velocity, stride length, step length, step time, and step width). Because of this, a random effects model was used to analyze the data. Sixteen studies (Demura and Uchiyama, 2009; Schabrun et al., 2014; Agostini et al., 2015; Jeon et al., 2015; Licence et al., 2015; Plummer et al., 2015; Strubhar et al., 2015; Krasovsky et al., 2018; Pau et al., 2018; Sirhan et al., 2018; Crowley et al., 2019; Feld and Plummer, 2019; Prupetkaew et al., 2019; Kim et al., 2020; Crowley et al., 2021; Krasovsky et al., 2021), including 29 comparisons, examined the effect of using a mobile phone on gait velocity. The result showed that mobile phone use significantly reduced participants’ gait velocity (SMD = −1.45; 95%CI: −1.66∼−1.24; I^2^ = 66%; *p* < 0.00001; Figure 2) and had a larger effect. The 21 comparisons from 12 studies (Demura and Uchiyama, 2009; Schabrun et al., 2014; Agostini et al., 2015; Jeon et al., 2015; Lim et al., 2015; Seymour et al., 2016; Krasovsky et al., 2018; Pau et al., 2018; Sirhan et al., 2018; Crowley et al., 2019; Kim et al., 2020; Krasovsky et al., 2021) also showed the same large effect of mobile phone use on stride length (SMD = −0.9; 95%CI: −1.19∼−0.60; I^2^ = 79%; *p* < 0.00001; Figure 3). Fifteen comparisons from seven studies (Jeon et al., 2015; Kao et al., 2015; Licence et al., 2015; Strubhar et al., 2015; Magnani et al., 2017; Prupetkaew et al., 2019; Kim et al., 2020) evaluated changes in step length. The pooled effect value was SMD = −1.01 (95%CI: −1.43∼−0.59; I^2^ = 82%; *p* < 0.00001; Figure 4), showing that mobile phone usage significantly decreased step length and had a large effect. The pooled effect value of twenty comparisons from eleven studies (Jeon et al., 2015; Kao et al., 2015; Licence et al., 2015; Strubhar et al., 2015; Magnani et al., 2017; Pau et al., 2018; Sirhan et al., 2018; Crowley et al., 2019; Prupetkaew et al., 2019; Kim et al., 2020; Tandon et al., 2021) was SMD = 0.77 (95%CI: 0.45–1.08; I^2^ = 78%; *p* < 0.00001; Figure 5), revealing that mobile phone usage showed a considerable increase in step time and that there was a large effect. Ten comparisons from six studies (Demura and Uchiyama, 2009; Kao et al., 2015; Lim et al., 2015; Seymour et al., 2016; Magnani et al., 2017; Lee and Lee, 2018) investigated step width, with a pooled effect value of SMD = 0.79 (95%CI: 0.34–1.24; I^2^ = 75%; *p* = 0.0006; Figure 6), showing that mobile phone usage significantly increased step width and had a medium effect.

**FIGURE 7:**
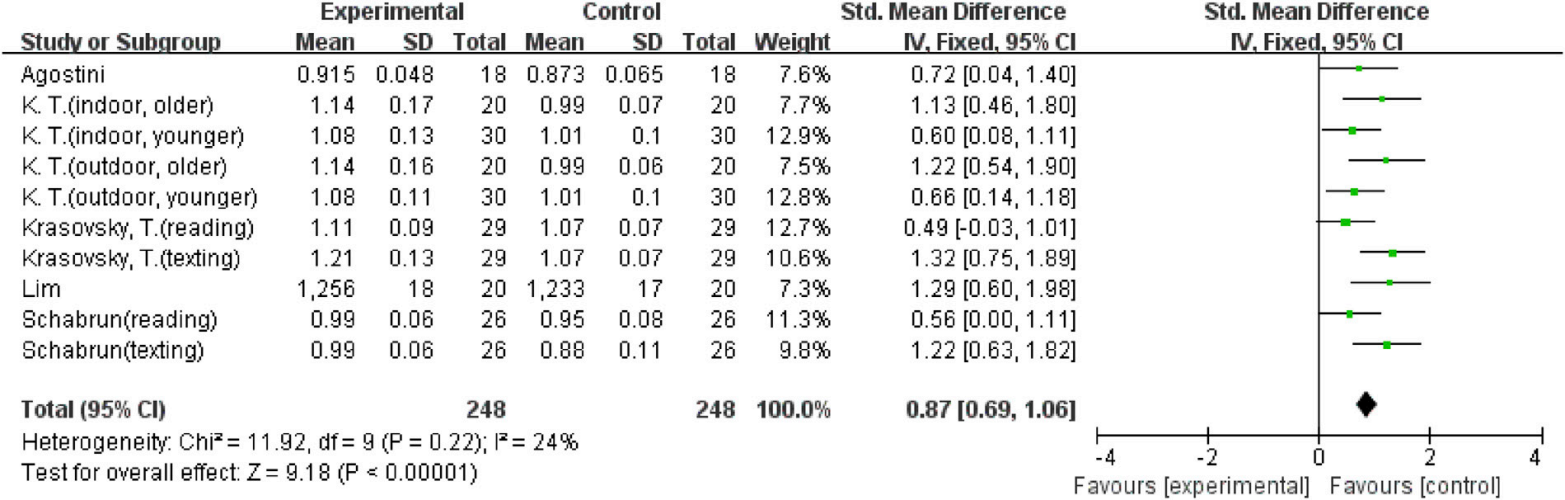
The effect of mobile phone use on stride time.

**FIGURE 8:**
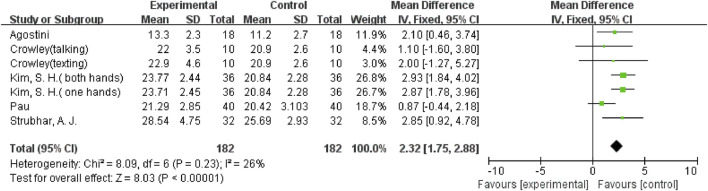
The effect of mobile phone use on double support (% GC).

**FIGURE 9:**
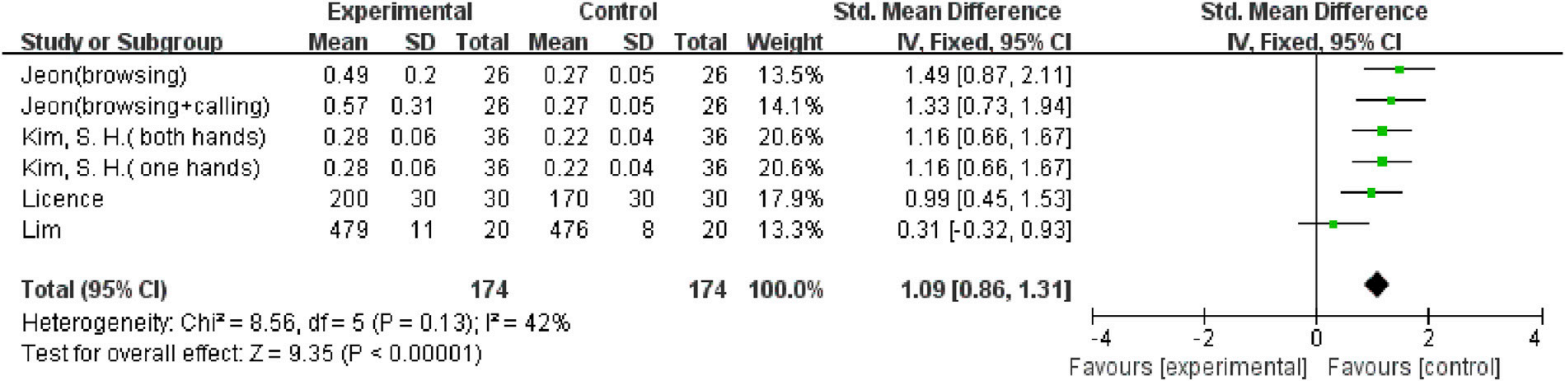
The effect of mobile phone use on double support time.

In addition, there were three outcome variables (stride time, double support (% GC), and double support time) with smaller heterogeneity; hence a fixed-effects model was used for the analysis. Five studies (Schabrun et al., 2014; Agostini et al., 2015; Lim et al., 2015; Krasovsky et al., 2018; Krasovsky et al., 2021), including ten comparisons, examined the effect of mobile phone use on stride time. The result showed that using a mobile phone significantly increased participants’ stride time (SMD = 0.87; 95%CI: 0.69–1.06; I^2^ = 24%; *p* < 0.00001; Figure 7) and had a larger effect. Seven comparisons from 5 studies (Agostini et al., 2015; Strubhar et al., 2015; Pau et al., 2018; Crowley et al., 2019; Kim et al., 2020) were examined for changes in double support (% GC), with a pooled effect value of MD = 2.32 (95%CI: 1.75–2.88; I^2^ = 26%; *p* < 0.00001; Figure 8), demonstrating that mobile phone usage significantly raised double support in participants and that there was a large effect. The six comparisons from 4 studies (Jeon et al., 2015; Licence et al., 2015; Lim et al., 2015; Kim et al., 2020) also showed the same large effect of mobile phone use on double support time (SMD = 1.09; 95%CI: 0.86–1.31; I^2^ = 42%; *p* < 0.00001; Figure 9).

### Subgroup analysis results

The participants’ ages and mobile phone use tasks were used as grouping factors in subgroup analyses to investigate the sources of gait velocity, stride length, step length, step time, and step width heterogeneity (Table 3; Table 4).

**TABLE 3:** Subgroup analysis of the effect of different mobile phone use tasks on gait parameters.

Outcomes	Intervention type	Number of comparisons pooled	Results of heterogeneity test (I^2^)	Effect model	Effect size (95% CI)	*p*-value
Gait velocity	texting	23	59	random	−1.56/(−1.78, −1.34)	*p* < 0.0001
	talking	2	28	fixed	−0.43 (−0.94, 0.09)	*p* = 0.1
	browsing	1	/	/	−1.71 (−2.35, −1.06)	/
	browsing + calling	1	/	/	−1.88 (−2.54, −1.22)	/
	reading	2	37	fixed	−0.87 (−1.27, −0.48)	*p* < 0.0001
Stride length	texting	14	81	random	−0.93 (−1.30, −0.56)	*p* < 0.0001
	talking	1	/	/	−0.54 (−1.44, 0.35)	/
	browsing	1	/	/	−1.82 (−2.47, −1.17)	/
	browsing + calling	1	/	/	−1.69 (−2.33, −1.05)	/
	reading	2	77	random	−0.69 (−1.50, 0.13)	*p* = 0.1
	dialing	2	0	fixed	−0.08 (−0.67, 0.51)	*p* = 0.78
Step length	browsing	1	/	/	−1.58 (−2.21, −0.95)	/
	browsing + calling	1	/	/	−1.69 (−2.33, −1.05)	/
	dialing	2	0	fixed	−0.19 (−0.89, 0.50)	*p* = 0.58
	texting	9	82	random	−1.26 (−1.79, −0.73)	*p* < 0.0001
	talking	1	/	/	−0.06 (−0.68, 0.56)	/
	listening	1	/	/	0.09 (−0.53, 0.71)	/
Step time	dialing	2	0	fixed	−0.21 (−0.91, 0.48)	*p* = 0.55
	texting	13	64	random	0.94 (0.65, 1.24)	*p* < 0.0001**
	talking	2	0	fixed	−0.18 (−0.69, 0.33)	*p* = 0.49
	browsing	1	/	/	1.42 (0.81, 2.03)	/
	browsing + calling	1	/	/	1.95 (1.28, 2.62)	/
	listening	1	/	/	−0.36 (−0.99, 0.26)	/
Step width	texting	4	0	fixed	0.41 (0.14, 0.68)	*p* = 0.003
	dialing	4	82	random	2.06 (0.66, 3.47)	*p* = 0.004
	talking	1	/	/	0.43 (−0.20, 1.06)	/
	listening	1	/	/	0.08 (−0.54, 0.70)	/

Note: / indicates that the number of included studies for subgroup analysis was not met.

**TABLE 4:** Subgroup analysis of the effect of mobile phone use on gait parameters in participants of different ages.

Outcomes	Age group	Number of comparisons pooled	Results of heterogeneity test	Effect model	Effect size (95% CI)	*p*-value
Gait velocity	younger	25	63	random	−1.39 (−1.60, −1.18)	*p* < 0.0001
	older	4	80	random	−2.17 (−3.22, −1.13)	*p* < 0.0002
Stride length	younger	18	88	random	−0.83 (−1.24, −0.41)	*p* < 0.0003
	older	3	34	fixed	−0.65 (−1.05, −0.25)	*p* = 0.002
Step length	younger	12	81	random	−0.87 (−1.30, −0.45)	*p* < 0.0003
	older	3	88	random	−1.79 (−3.58, 0.00)	*p* = 0.05
Step time	younger	17	78	random	0.70 (0.38, 1.03)	*p* = 0.0001**
	older	3	85	random	1.25 (−0.21, 2.71)	*p* = 0.09
Step width	younger	8	74	random	0.62 (0.15, 1.09)	*p* = 0.01
	older	2	0	fixed	1.55 (0.82, 2.28)	*p* < 0.0003

In the subgroup analysis of the mobile phone use task (Table 3), the heterogeneity among studies for gait velocity, and step time reduced, suggesting that the mobile phone use task may be an important source of heterogeneity. For the resource-intensive tasks (texting and reading task), the study results showed that using a mobile phone significantly reduced participants’ gait velocity (texting task (Demura and Uchiyama, 2009; Schabrun et al., 2014; Agostini et al., 2015; Licence et al., 2015; Plummer et al., 2015; Strubhar et al., 2015; Krasovsky et al., 2018; Pau et al., 2018; Sirhan et al., 2018; Crowley et al., 2019; Feld and Plummer, 2019; Prupetkaew et al., 2019; Kim et al., 2020; Crowley et al., 2021; Krasovsky et al., 2021): SMD = −1.56; 95% CI: −1.78∼−1.34; I^2^ = 59%; *p* < 0.0001; reading task (Strubhar et al., 2015; Krasovsky et al., 2021): SMD = −0.87; 95% CI: −1.27∼-0.84; I^2^ = 37%; *p* < 0.0001), and step time (texting task (Sirhan et al., 2018; Prupetkaew et al., 2019; Tandon et al., 2021): SMD = 1.15; 95% CI: 0.61–1.68; I^2^ = 64%; *p* < 0.0001). However, the small resource-intensive tasks (calling, talking and dialing task) on gait velocity (calling task (Crowley et al., 2019; Crowley et al., 2021): SMD = −0.43; 95% CI: −0.94–0.09; I^2^ = 28%; *p* = 0.1), and step time (dialing task (Kao et al., 2015): SMD = −0.21; 95% CI: −0.91–0.84; I^2^ = 0%; *p* = 0.55; talking task (Magnani et al., 2017; Crowley et al., 2019): SMD = −0.18; 95% CI: −0.69–0.33; I^2^ = 0%; *p* = 0.49) had no significant effects. In addition, the stride length, step length, and step width heterogeneity were not significantly reduced, but the degree of impact on different indicators still varies between tasks.

In the subgroup analysis of the participant’s age (Table 4), the heterogeneity among studies for step width reduced, suggesting that the participant’s age may also be an important source of heterogeneity. The results showed that using a mobile phone significantly increased step width in older and younger participants (older (Kao et al., 2015; Seymour et al., 2016): SMD = 1.55; 95% CI: 0.82 to 2.28; I^2^ = 0%; *p* < 0.0003; younger (Demura and Uchiyama, 2009; Kao et al., 2015; Lim et al., 2015; Seymour et al., 2016; Magnani et al., 2017; Lee and Lee, 2018): SMD = 0.62; 95% CI: 0.15 to 1.09; I^2^ = 74%; *p* = 0.01). In addition, the gait velocity, stride length, step length, and step time heterogeneity were not significantly reduced, but the degree of impact on different indicators also still varies between tasks.

### Sensitivity analysis

Sensitivity analyses on the outcomes (gait velocity, stride length, step length, step time, and step width) that included more than ten items for comparison were performed by removing each comparison to explore the sources of heterogeneity further.

The results have shown that, for the step width outcome, the pooled effect value of SMD = 0.43 (95% CI: 0.21–0.65; I^2^ = 11%; *p* = 0.0001) after excluding the two comparisons from Seymour (Seymour et al., 2016) did not change, but its heterogeneity was significantly lower, suggesting that this study may be a source of heterogeneity. The pooled effect values of the remaining outcomes were consistent with the original analysis, and the single study had little effect on the pooled results, indicating that the overall results of this study were reliable.

## Publication bias test

Publication bias was examined using Egger’s test for outcomes (gait velocity, stride length, step length, and step width), for which more than ten items were included in the comparison. The study results are shown in Table 5. The results showed no publication bias (*p* > 0.05) for all outcomes except for the step width. The trim and fill method further evaluated the effect of publication bias on the step width. The results showed that the pooled effect value and significance did not change before and after trim and fill, indicating that the results of this study were reliable.

**TABLE 5:** Publication bias test for gait parameters.

Outcomes	t-value	*p*-value
Gait velocity	−1.74	0.094
Stride length	0.67	0.512
Step length	−0.66	0.522
Step width	4.28	0.003

## Discussion

To our knowledge, this is the first meta-analysis to analyze the effectiveness of mobile phone use on pedestrians’ gait and to compare differences in the effects of mobile phone tasks and the participant’s age. In this meta-analysis, we reviewed 22 studies, including 592 participants. Our results found that mobile phone usage decreased pedestrians’ gait velocity, stride length, and step length and increased their step time, stride time, step width, double support time, and double support (% GC). Interestingly, the subgroup analysis results showed differences in the effects of mobile phone use tasks and participants’ age on gait indicators.

Using a mobile phone can significantly affect pedestrian gait parameters resulting in reduced walking ability. In light of the finite capacity scheduling (Kahneman, 1973), a previous review study (Tan et al., 2022) found that using a mobile phone while walking decreases the percentage of motion control resources, increases cognitive load, and raises motion control challenges, all of which lead to decreased pedestrian walking ability (Lacour et al., 2008) and is reflected in the decreased gait velocity, stride length, and step length, as well as the rise in step width, step time, and double support time. All of them are generally consistent with the results of this study. To reduce the potential risks associated with reduced walking ability (e.g., fall risk), pedestrians often exhibit a more “cautious” movement pattern during mobile phone use to reduce the difficulty of movement execution, which may be the main reason for the changes in gait. It is worth noting that before this study, the results of studies on step length (Magnani et al., 2017), step time (Kao et al., 2015), cadence (Magnani et al., 2017), and stride length (Lim et al., 2015) were unclear. In this meta-analysis, the results of several studies were statistically pooled, considering data consistency, to determine the impact of using a mobile phone on the above indicators. The results showed that the use of mobile phones significantly decreased pedestrian step length and stride length and increased pedestrian step time, and all the results had a large effect. Compared with previous studies, the present meta-analysis not only increased the total sample size but also reduced the selection bias of the study population, thus compensating for the poor statistical efficacy that occurred in a single study. Therefore, the findings of the present meta-analysis may be more comprehensive, reliable, and persuasive.

In contrast to small resource-intensive tasks (calling, talking, and dialing tasks), resource-intensive tasks (texting and reading) significantly reduce some walking ability. For the cognitive load of mobile phone tasks, a narrative review (Tan et al., 2022) has shown that texting and reading were resource-intensive tasks, and the rest were small resource-intensive tasks. On the one hand, the cognitive load in different mobile phone tasks differs (Krasovsky et al., 2017). Compared to dialing, talking, and calling tasks, texting and reading tasks require more coordinated cooperation of sensory organs to complete action tasks efficiently (Neider et al., 2010; Galván et al., 2013). Their occupancy of cognitive resources will be significantly higher than dialing, talking, and calling tasks (Tan et al., 2022), which may be the main reason for the differences in step speed, step length, and step time between different mobile phone use tasks. On the other hand, when using a mobile phone to perform texting or reading tasks while walking, pedestrians need to pay more attention to the phone screen, reducing their ability to perceive their surroundings and control movements (Krasovsky et al., 2017). Due to self-protection strategies, pedestrians will actively reduce their gait velocity, step length, and cadence and increase their step time to prevent dangerous accidents. In essence, they may all be considered as a result of different levels of resource occupancy.

Interestingly, our results also showed that different resource-intensive tasks affect the stride length differently. In contrast, small resource-intensive tasks have the same effect on the step width. Firstly, the texting task is more like an integration of the reading and typing action tasks (Krasovsky et al., 2021), and its cognitive resource utilization will be greater than the reading task (Tan et al., 2022). This may be why the texting task significantly reduces the stride length while the reading task does not affect it. Secondly, our study also found that both resource-intensive tasks (texting tasks) and small resource-intensive tasks (dialing tasks) significantly increase the step width. Previous studies (Young and Dingwell, 2012) have suggested that changes in step width are a compensation strategy of stabilization employed by participants to compensate for deficits in motor control by increasing lateral stability. From the current results, pedestrians with even small cognitive loads during walking may adopt stability compensation to reduce the risk of accidents; however, further studies are needed to explore it due to the small number of studies included in the step width. In addition, a subgroup analysis of the influence of the dialing task on gait velocity was not conducted in this study due to the insufficient number of included studies. However, previous studies (Seymour et al., 2016; Crowley et al., 2021) have shown that the reduction in gait velocity may have a greater effect on stride length, step length, and step time than the dialing task. Therefore more studies should be conducted to clarify the underlying mechanism in the future.

In contrast to younger participants, the step length and step time of older participants were not affected by mobile phone use. Previous studies (Tan et al., 2022) have suggested that using a mobile phone affects older participants more than younger participants, but our study found the opposite. Older participants’ attentional allocation and action execution abilities do not necessarily decline with age. However, they may show a positive trend with age, which may be a significant reason for older participants’ step length and step time being less affected by cell phones (Spreng and Turner, 2019; Verissimo et al., 2022). On the other hand, this meta-analysis contained just two studies of older individuals, totaling 21 participants. The limited sample size of the studies, and the diverse experiment settings, may have contributed to the differences in the outcomes, and further research is needed in the future.

Although we comprehensively assessed all eligible studies, it still has some limitations. First, the number of studies is low for older people, which limits the external validity of the results. Second, the cross-sectional design of the included studies made it difficult to assess the quality of the evaluation. The Downs and Black checklist was determined to be the best fit for this meta-analysis. This scale, however, is less precise than the scale employed in the interventional study. We, therefore, thoroughly explored the sources of heterogeneity through subgroup analysis. Future studies should utilize randomized controlled experiments with large sample sizes to optimize the experimental testing procedure and conduct studies based on various influencing factors (e.g., mobile phone task, gender, age, different environments setting) to validate the results of this study. Third, some included studies were multi-arm trials, and there may be intra-study variation due to multiple data entries from the same study. Although we used sensitivity analysis to test the reliability of our findings, future studies could consider using multilevel models to address such issues.

## Conclusion

In summary, the current systematic review and meta-analysis proved that using a mobile phone by pedestrians while walking might significantly affect their gait resulting in reduced walking ability. Interestingly, participants’ age and mobile phone use tasks had different effects on walking performance. Resource-intensive tasks (texting and reading tasks) significantly reduced gait velocity, and step time, while small resource-intensive tasks (calling, talking, and dialing tasks) had no effect. In contrast to young adults, mobile phone use did not affect step length and step time in older participants.

## Data Availability

The original contributions presented in the study are included in the article/supplementary material, further inquiries can be directed to the corresponding authors.
